# The impact of equity factors on receipt of timely appropriate care for children with suspected malaria in eastern Uganda

**DOI:** 10.1186/s12889-021-11908-0

**Published:** 2021-10-16

**Authors:** David Humphreys, Joan Nakayaga Kalyango, Tobias Alfvén

**Affiliations:** 1 Department of Global Public Health, Karolinska Institutet, Stockholm, Sweden; 2 Clinical Epidemiology Unit, Makerere University College of Health Sciences, Kampala, Uganda; 3 Department of Pharmacy, Makerere University College of Health Sciences, Kampala, Uganda; 4 Sachs’ Children and Youth Hospital, Stockholm, Sweden

**Keywords:** Equity, Uganda, Care-seeking, Malaria, Fever, Child mortality

## Abstract

**Introduction:**

Malaria accounts for more than one-tenth of sub-Saharan Africa’s 2.8 million annual childhood deaths, and remains a leading cause of post-neonatal child mortality in Uganda. Despite increased community-based treatment in Uganda, children continue to die because services fail to reach those most at risk. This study explores the influence of two key equity factors, socioeconomic position and rurality, on whether children with fever in eastern Uganda receive timely access to appropriate treatment for suspected malaria.

**Methods:**

This was a cross-sectional study in which data were collected from 1094 caregivers of children aged 6–59 months on: illness and care-seeking during the previous two weeks, treatment received, and treatment dosing schedule. Additional data on rurality and household socioeconomic position were extracted from the Iganga-Mayuge Health and Demographic Surveillance Site (HDSS) database. A child was considered to have received prompt and appropriate care for symptoms of malaria if they received the recommended drug in the recommended dosing schedule on the day of symptom onset or the next day. Unadjusted and adjusted logistic regression models were developed to explore associations of the two equity factors with the outcome. The STROBE checklist for observational studies guided reporting.

**Results:**

Seventy-four percent of children had symptoms of illness in the preceding two weeks, of which fever was the most common. Children from rural households were statistically more likely to receive prompt and appropriate treatment with artemisinin-combination therapy than their semi-urban counterparts (OR 2.32, CI 1.17–4.59, *p* = 0.016). This association remained significant following application of an adjusted regression model that included the age of the child, caregiver relationship, and household wealth index (OR 2.4, *p* = 0.036). Wealth index in its own right did not exert a significant effect for children with reported fever (OR for wealthiest quintile = 1.02, CI 0.48–2.15, *p* = 0.958).

**Conclusions:**

The findings from this study help to identify the role and importance of two key equity determinants on care seeking and treatment receipt for fever in children. Whilst results should be interpreted within the limitations of data and context, further studies have the potential to assist policy makers to target inequitable social and spatial variations in health outcomes as a key strategy in ending preventable child morbidity and mortality.

## Introduction

Despite significant reductions in global child mortality achieved during the past quarter of a century, almost five and a half million children under the age of five continue to die around the world every year, largely from preventable causes [1, 2]. If present trends continue, modelling suggests 3.8 million children will still die unnecessarily in the year 2030 [3]. Over half of these deaths occur in sub-Saharan Africa, where mortality rates some fourteen times greater than that of high income regions are driven by low intervention coverage due to weak delivery systems, poor linkages with maternal health programmes, and gaps in the continuum of care, among other factors [2, 4, 5].

In Uganda, leading causes of childhood mortality closely mirror those of sub-Saharan Africa as a whole. Despite concerted control efforts as part of the Ministry of Health’s strategic plan, malaria continues to account for over one tenth of all childhood deaths [6]. Timely access to good health care is crucial to enable prompt diagnosis and the commencement of effective antimalarial treatment. However, despite upscaling access to rapid-diagnostic testing (RDT) and artemesinin-based combination therapies (ACT) children continue to die from malaria in settings where services are fragmented and fail to reach those most at risk [7].

Building upon the Integrated Management of Childhood Illness (IMCI) strategy which started in Uganda in 1996, a policy promoting community-based testing and treatment of malaria initially through the home-based management of fever was established in 2002. The aim of this strategy was to improve access to health care for the rural poor who traditionally have lacked financial resources for transportation to distant health facilities [8]. In 2010, this strategy was expanded to include management of pneumonia and diarrhoea under the integrated community case management strategy (iCCM). The principles of iCCM have scaled up community-level care delivery where it is most needed, particularly in rural areas.

However, even when basic primary health care is available, care-seeking is influenced by multiple other factors including (but not limited to): recognition of a child’s symptoms by the caregiver, perception of symptom severity, and a diverse array of barriers beyond the caregiver’s control. These barriers, such as the cost of accessing and purchasing treatment and distance to the nearest point of care, may greatly impact decisions regarding care-seeking and so perpetuate existing inequalities in health outcomes. Access to health services remains a complex notion, however this study employed a framework which considers access as the continuum of identifying health care needs, seeking healthcare services, obtaining or utilising those services, and being offered and receiving services which match those needs [9].

The central role equity plays in improving health and development outcomes is emphasised in both the UN’s 2030 Agenda for Sustainable Development and associated Sustainable Development Goals (SDGs). Goal ten specifically provokes member states to work together to “reduce inequality within and between countries” [10]. At a country level, delivery of important health interventions often remains poorest where the needs are greatest [11]. National averages thus oft belie important inequities, with significant disparities characterising those receiving access to local health services [12]. Addressing inequality with regard to child health is an ongoing and increasing focus for research and policy makers alike [13–15].

Given the need to understand the impact of health determinants upon the goal of achieving more equitable health outcomes, this study aimed to explore the influence of socioeconomic position and rurality on care-seeking for children aged 6–59 months with fever in eastern Uganda.

## Methods

### Study design and setting

A cross-sectional study was conducted in eastern Uganda in the area covered by the Iganga-Mayuge Health and Demographic Surveillance Site (HDSS), a collaboration between the Makerere University School of Public Health and the Karolinska Institutet. The HDSS covers a total area of 3931 km^2^ and straddles the border between the two districts of Iganga and Mayuge, approximately 120 km east of the capital Kampala. Consisting of 65 villages, the region is predominantly rural with a largely subsistence-agricultural economy. The total population of the area is approximately 79,910 [16], of whom approximately 17% are children under the age of five years [17].

Data were attained using a household questionnaire in a setting where a cluster Randomized-Controlled Trial (cRCT) was ongoing [18]. The purpose of this cRCT study was to compare community-based management of malaria alone to the integrated community-based management of malaria and non-severe pneumonia [19–22]. Under the cRCT, the HDSS was divided into intervention and control areas. In both areas, Community Health Workers (CHWs) provided care for malaria symptoms among children aged 6–59 months. The CHWs considered children with fever to have malaria. Additionally, CHWs in the intervention area provided care for pneumonia symptoms.

### Sample size estimation

The sample size for the study (1094) was estimated for the primary objective that compared prompt and appropriate treatment among sick children in the intervention and control areas of the cRCT that was ongoing in the HDSS. It was considered sufficient to address the objective of the current study comparing prompt and appropriate treatment among children in rural and urban areas based on formula for comparison of two proportions. Assumptions included: 5% level of significance, 80% power, ratio of children in rural to urban of 4:1, estimated proportion of children with prompt and appropriate treatment from rural and urban areas of 61 and 83% respectively. The latter was estimated from documented proportions of children in rural and urban areas with entitlements to health care [23]. These assumptions gave 207 children with symptoms of malaria which translated to 959 children when design effect of 2, 54% of children ill in the previous two weeks [24], and 20% missing data were considered.

### Data collection

The household questionnaire was administered by trained research assistants to 1095 caregivers of children aged between 6 and 59 months. All participating caregivers gave informed consent prior to inclusion in the study.

Information collected in the questionnaire pertained to whether a child under their care had been unwell in the previous two weeks and, if so, symptomatology, care-seeking behaviour, and any treatment received. Significant efforts were employed during interviews to improve the validity of treatment reporting by caregivers. Examples included the use of medicine posters showing drugs commonly used in the area to assist correct identification of treatment received, and cross-checking with medication packages to confirm the drug and dose prescribed to the child, where these were available.

Data relating to both the location of households (rural versus semi-urban) and their corresponding asset-wealth quintiles were extracted from the Iganga-Mayuge HDSS, imported, and merged with the existing questionnaire dataset using unique identifiers. Composite variables were constructed to operationalize the outcome of interest, specifically whether the child in question received prompt and appropriate care for fever. This outcome measure required satisfactory (i) commencement of a recommended antimalarial drug based on local guidelines, (ii) within 24 h of symptom onset, and (iii) at an appropriate dose, (iv) frequency and (v) duration.

**Fig. 1:**
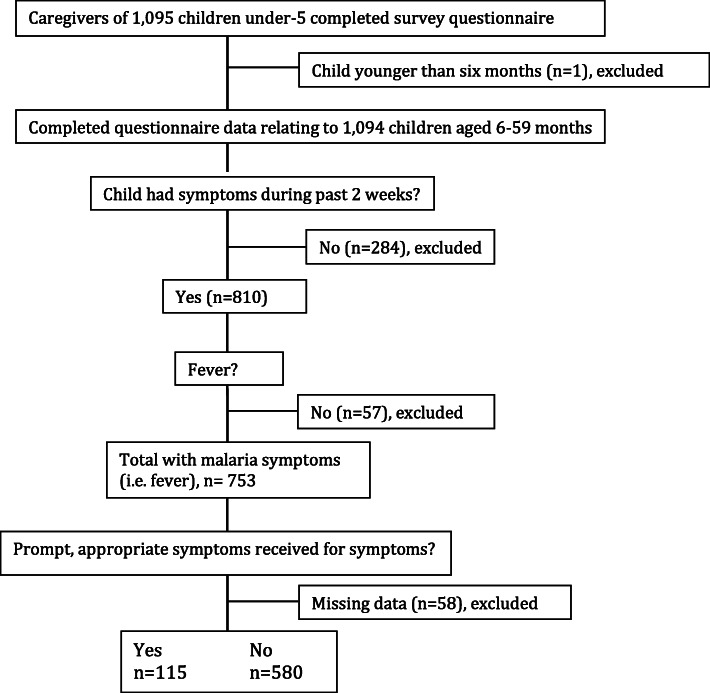
Algorithm of sampling framework

Figure 1 outlines the sampling framework for this study. Initially, 1400 children aged 6–59 months were sampled by simple random sampling from the HDSS database, stratified by arm of the cRCT (700 from the intervention and 700 from the control areas). Among these, caregivers of 1095 children completed the survey questionnaire. One response was excluded as the child in question lay outside the target age range for this sub-study (being 6 months to 5 years). Children who hadn’t been unwell in the two weeks prior to interview (*n* = 284), and those with symptoms not relating to malaria (i.e. no fever, *n* = 57), were also excluded. A period of two weeks was considered suitable for analysis since longer periods may have resulted in recall bias which may have impacted the quality of the data associated with care sought and treatment received.

The Iganga-Mayuge HDSS undertakes regular updates of household wealth status based on a modified asset score similar to that employed by the Demographic and Health Surveys (DHS) Program [25]. A composite score is generated using principal components analysis and divided into quintiles. Additionally, households within the HDSS are classified as either rural or semi-urban, based on population size and proximity to regional centres. Both classifications were maintained and data were matched with the corresponding households of the caregivers participating in the questionnaire survey.

Appropriate treatment of subjective fever, the sole symptom considered for a clinical diagnosis of malaria, was defined as the child receiving an appropriate antimalarial (based on national guidelines), at the right frequency and dose, for the correct duration [26]. Prompt commencement of treatment necessitated drug commencement on the same or next day following the recognition of signs or symptoms in the child. This definition has been applied previously as a temporal measure of 24 h in a setting and culture where watches are seldom worn [24, 27]. A total of 58 cases were excluded from analysis due to missing data which led to a lack of clarity whether prompt, appropriate treatment was received.

### Data analysis

Stata 13 (StataCorp., TX, USA) was employed to perform analyses for this study. Baseline characteristics of the study population were summarized using descriptive statistics, with bivariate analyses being undertaken using Pearson’s chi-squared test, Fisher’s exact test, and the Kruskal-Wallis rank sum test. Variables that had a *p*-value of < 0.2 on bivariate analysis were included in the adjusted logistic regression model. Rate differences and ratios were calculated for analysis of wealth index and rurality against prompt and appropriate access to treatment.

Reference groups were defined to either reflect the most common scenario (for example, the relationship of caregiver to child being mother), or those potentially most disadvantaged (for example, rural location, lowest wealth quintile) in order to observe for protective effect

## Results

### Characteristics of study population

After excluding those children who didn’t have symptoms of illness within the two weeks preceding questionnaire completion, those with symptoms other than fever, and missing information precluding definitive outcome measurement, data from a total of 695 caregivers were included for analysis. The sample characteristics of these caregivers and their children are presented in Table 1. The average age of children was 33.8 months. Mothers were the primary caregiver in three-quarters (75%) of cases. Eighty-three percent of questionnaire respondents resided in rural areas. The proportion of the sample belonging to various wealth quintiles varied from 22% in the middle quintile to 10% in the highest quintile. Information pertaining to wealth status was missing for 14% of the survey population.

**Table 1:** Socioeconomic and demographic characteristics of study population

Characteristics	Total	
	n	%
Total number of completed questionnaires for children aged 6–59 months with fever during the past 2 weeks	695	100
**Age of child**		
Mean (S.D.), months	33.8 (14.6)	
6 months – 1 year	62	8.9
1–3 years	297	42.7
3–5 years	333	47.9
Missing	3	0.4
TOTAL	695	100
**Sex of child**		
Female	362	52.1
Male	333	47.9
TOTAL	695	100
**Caregiver relationship to child**		
Mother	518	74.5
Father	69	9.9
Aunt	10	1.4
Uncle	1	0.1
Grandmother	9	1.3
Grandfather	57	8.2
Other*	8	1.2
Missing	23	3.3
TOTAL	695	100
**Wealth index (quintiles)**		
Lowest	115	16.6
2nd	129	18.6
Middle	150	21.6
4th	130	18.7
Highest	72	10.4
Missing	99	14.2
TOTAL	695	100
**Rurality**		
Semi-urban	115	16.6
Rural	580	83.4
TOTAL	695	100.00

* Other: sister (3), sister-in-law (1), step-mother (4)

### Reporting of symptoms by caregivers

In the two weeks prior to the questionnaire being completed, almost three quarters (74%) of all caregivers surveyed reported their child had symptoms of illness (not limited to malaria). Overwhelmingly, fever was the most common symptom reported (93% of those unwell), either in isolation or in conjunction with other symptoms.

Symptom reporting was highest among caregivers from households in the poorest wealth quintile, 80% of whom reported their child had been unwell during the preceding two weeks. Proportionately higher rates of illness were reported among children living in rural areas (76%) compared to semi-urban dwellers (68%).

### Timely and appropriate care for symptoms of malaria

Table 2 presents bivariate and multivariable analyses for children with fever. Due to the composite nature of the outcome variable, missing data meant only 695 observations were included in the final statistical analysis.

**Table 2:** Bivariate and adjusted analyses for children with symptoms of malaria

Socio-economic and demographic characteristics	Child received timely and appropriate treatment for symptoms of malaria?									
	Total		Yes		No		Crude		Adjusted	
	n	%	n	%	n	%	OR (95% C.I.)	P-value	OR (95% C.I.)	*P*-value
n	695	100.0								
**Age of child (months)**										
Mean (95% C.I.)	33.80 (32.71–34.88)		31.30 (28.51–34.10)		34.29 (33.12–35.47)			**0.044**	0.99 (0.97–1.00)	0.082
**Sex of child**										
Female	362	52.1	56	48.7	306	52.8	1	Ref		
Male	333	47.9	59	51.3	274	47.2	1.18 (0.79–1.76)	0.426		
TOTAL	695		115		580					
**Caregiver relationship to child**										
Mother	518	74.5	79	68.7	439	75.7	1	Ref	1	Ref
Father	69	9.9	14	12.2	55	9.5	1.41 (0.75–2.67)	0.283	1.55 (0.79–3.06)	0.205
Aunt	10	1.4	4	3.5	6	1.0	7.12 (1.02–13.43)	**0.046**	9.11 (1.85–44.89)	**0.007**
Uncle	1	0.1	0	0.0	1	0.2				
Grandfather	57	8.2	12	10.4	45	7.8	1.48 (0.75–2.93)	0.257	1.39 (0.65–2.94)	0.396
Grandmother	9	1.3	0	0.0	9	1.6				
Other*	8	1.2	1	0.9	7	1.2	0.79 (0.10–6.54)	0.830	0.97 (0.11–8.47)	0.980
Missing	23	3.3	5	4.4	18	3.1				
TOTAL	695		115		580					
**Wealth index (quintiles)**										
Lowest	115	16.6	22	19.1	93	16.0	1	Ref	1	Ref
2nd	129	18.6	21	18.3	108	18.6	0.82 (0.43–1.59)	0.560	0.81 (0.41–1.60)	0.543
Middle	150	21.6	26	22.6	124	21.4	0.89 (0.47–1.66)	0.707	0.86 (0.44–1.67)	0.658
4th	130	18.7	15	13.0	115	19.8	0.55 (0.27–1.12)	**0.101**	0.58 (0.28–1.23)	0.156
Highest	72	10.4	14	12.2	58	10.0	1.02 (0.48–2.15)	0.958	1.42 (0.64–3.17)	0.389
Missing	99	14.2	17	14.8	82	14.1				
TOTAL	695		115		580					
**Rurality**										
Semi-urban	115	16.6	10	8.7	105	18.1	1	Ref	1	Ref
Rural	580	83.4	105	91.3	475	81.9	2.32 (1.17–4.59)	**0.016**	2.40 (1.06–5.45)	**0.036**
TOTAL	695		115		580					

*Other: sister (3), sister-in-law (1), step-mother (4)

With mothers being the predominant caregiver surveyed and employed as the reference group, the role of aunty as caregiver demonstrated a protective relationship (OR 7.12, CI 1.02–13.43, *p* = 0.046). This association remained significant following application of an adjusted regression model that also included the age of the child, rurality, and household wealth index (OR 9.11, CI 1.85–44.89, *p* = 0.007), albeit with a very wide confidence interval.

Wealth index in its own right did not exert a significant effect for children with reported isolated fever (OR for wealthiest quintile = 1.02, CI 0.48–2.15, *p* = 0.958).

In terms of rurality, data demonstrated an association whereby children from rural households were statistically more likely to receive prompt and appropriate care for fever than their semi-urban counterparts (OR 2.32, CI 1.17–4.59, *p* = 0.016). This association remained significant following application of the adjusted regression model described above (OR 2.4, *p* = 0.036).

### Specific equity range measures

Calculation of a rate ratio demonstrated no difference among children presenting with fever: that is, those from the highest wealth quintile were no less likely to receive prompt and appropriate care than those in the lowest.

For rurality however, being from a rural household appeared protective: children from semi-urban households were almost half (0.45 times) as likely to receive timely appropriate care than their rural counterparts.

## Discussion

### Reporting of symptoms

Nearly three quarters (74%) of caregivers reported their child had been unwell during the two weeks prior to being surveyed – 69% of those surveyed with fever being the chief symptom. This represents a high proportion, but is consistent with previous data from Uganda [28]. The 2016 Ugandan Demographic and Health Survey (DHS) reported 66% of children under five in the Busoga region (which includes Iganga and Mayuge districts) had fever during the preceding two weeks; the most recent Malaria Indicator Survey (MIS) estimated a preceding two-week fever prevalence of 47% among children from the same region [29].

The higher rates of symptom-reporting for children from both rural and the poorest households remains consistent with evidence that both demographic cohorts experience a higher burden of disease than that of their urban, wealthier counterparts [30, 31]. Enhanced community sensitization, both as a result of being located in an HDSS, and the added data collection associated with the larger on-going cRCT, may also have played a role in increasing awareness of and vigilance in recognising and reporting symptoms.

### The relationship of caregivers

A disproportionate burden of caregiving was placed on female figures within the household, principally mothers, who accounted for almost three-quarters (74.5%) of caregivers surveyed. This is consistent with previous country-level gender assessments, which continue to recognise the large proportion of unpaid “care” work in the household, including child and elder care, undertaken by women [32].

The statistically significant protective role aunts appear to play as primary caregivers was an interesting albeit unanticipated outcome. Whilst not an equity factor per se, the small sample size and wide confidence intervals suggest further exploration may be warranted if robust conclusions about the practical significance of this relationship are to be made.

### The role of wealth

Previous studies have demonstrated that those in the poorest wealth quintile experience greatest delays in accessing care [26]. Even in settings where “free” health services exist, poverty is associated with a delay in care-seeking. This study, whilst not demonstrating a significant association, sought to extend such previous studies beyond care-seeking to encompass the goal of receiving timely appropriate treatment.

This lack of an observed effect is likely to be multifactorial. Small sample size, missing data, and inadequate power may have contributed. The additional fact that the broader HDSS, wherein the quintiles are formulated, is a generally homogenous socioeconomic area (that is, the absolute difference in asset-wealth from the poorest to the least poor is not that marked) may also play a role in buffering the socioeconomic gradient.

So too, the effect of Community Health Workers (CHWs) which have been employed in the area for some time, may have served to reduce the effect of wealth-related inequality – however further targeted research would be necessary to adequately evaluate this hypothesis.

### The role of rurality

The influence of rural or semi-urban location was significant for children with symptoms of malaria. Children from rural households with fever were *more* likely to receive prompt and appropriate malaria treatment than their peri-urban counterparts. One possible explanation for this result may relate to the historical tendency for CHWs – and people living in endemic areas – to associate fever with malaria. As a result, awareness of fever as a symptom is high in rural areas where transmission rates are highest and a historical tendency emphasising presumptive treatment has been challenging to modify at both community and health system levels [33, 34]. Better CHW coverage in rural areas likely also played a significant role: whilst urban areas have higher drug shop penetration, some of these may provide inappropriate treatment not aligning with recommended antimalarial therapy.

There may also be a hidden association linking cases of children from wealthier and urban settings. Small numbers in both the highest wealth index group (Q5 *n* = 14) and the urban group receiving care (*n* = 10) mean any parallel effect could be due to the same sample group, a recognised limitation to wealth stratification alongside geographical location [35]. As with the findings from wealth stratification, increased sample size and study power would clarify if any underlying association was present.

### Methodological limitations

Important methodological and data limitations impacted upon the analysis and findings of this study. First, a lack of available data concerning other equity and demographic variables including, for example, caregiver education status and birth order, limited the study’s scope significantly. Whilst extensive evidence has highlighted the importance of rurality and socioeconomic status on both childhood mortality and coverage of health interventions [28, 36], consideration of additional equity factors would have facilitated a more comprehensive examination. Second, owing largely to the composite nature of the outcome variables, missing data significantly limited the sample size for analysis. In combining several individual variables to form composite outcome variables, missing data for any of the individual variables (e.g. whether the correct dosage was administered or the full course of treatment was completed) was amplified and served to annul the composite. We made the choice to define such data as missing to avoid drawing any false conclusions. This resulted in about 8% (*n* = 58) of the 753 eligible children to be excluded from the analysis. Whilst this was a limitation of the dataset available, future researchers would be advised to factor such challenges into study design and planning wherever possible. Finally, the collection of data in circumstances where a cRCT was ongoing may have led to increased public awareness, influencing symptom recognition and care seeking behaviour among caregivers at the time of data collection.

Rapid advances in point-of-care malaria diagnostics have also made positive impacts in low-resource settings, including Uganda. Since 2010, WHO has recommended confirmation of all suspected malaria cases by microscopy or rapid diagnostic test (RDT) before treatment [37]. Indeed, the IMCI guidelines for high-risk malaria settings promote testing wherever available (38). Thus, with time and increased resourcing, widespread empiric treatment based on symptoms alone will reduce in favour of targeted treatment for those parasitological-positive cases identified, even in hyper-endemic settings.

## Conclusion

While these results should be interpreted within the limitations of the data and context described above, the associations identified in this study warrant further investigation. The significant role of rurality may be influenced by several factors, including (but not limited to) the important work of CHWs in improving health-literacy regarding symptom identification and facilitating early access to care.

Further mixed methods-oriented research may compliment quantitative data (for example that of Demographic and Health Surveys) to identify further barriers and enablers impacting upon inequities. Robust findings from such studies have the potential to improve access to essential child health services, both in dedicated facilities and within the community.

Although the findings from this study may not necessarily be generalized outside of this context, Uganda, like many other low-income countries, continues to experience significant variations in coverage levels for important child health interventions. Rurality and socioeconomic position undoubtedly impact upon a child’s access to timely appropriate care and help to explain inequitable social and spatial variations in health outcomes. Whilst not available for inclusion in this study, additional equity variables, such as caregiver education status and ethnic grouping, would add value. Whilst beyond the scope of this study, gender assessments have already explored the important roles of women as primary caregivers in traditional patriarchal societies.

Greater understanding of the role and importance of these health determinants will assist policy-makers to better target early intervention programmes, whether in relation to enhancing health literacy, improving access to care, or other measures which positively discriminate in order to promote equity of health outcomes. Addressing these coverage gaps by targeting those most in need should remain a key strategy in the global effort to end preventable child morbidity and mortality. Such action will be critical if countries like Uganda are to meet the targets of reducing preventable child mortality and intra-country inequality as part of their commitments to the SDGs.

## Data Availability

The datasets analysed during the current study are available from the corresponding author on reasonable request.
